# Computer-Based Attention-Demanding Testing Unveils Severe Neglect in Apparently Intact Patients

**DOI:** 10.3233/BEN-2012-129005

**Published:** 2013

**Authors:** M. Bonato, K. Priftis, C. Umiltà, M. Zorzi

**Affiliations:** ^1^Department of General PsychologyUniversity of PadovaPadovaItaly; ^2^Laboratory of NeuropsychologyIRCCS San CamilloLido-VeniceItaly

**Keywords:** Neglect, dual-task, spatial awareness, attentional resources, cognitive load, right hemisphere damage, neuropsychological assessment

## Abstract

We tested a group of ten post-acute right-hemisphere damaged patients. Patients had no neglect according to paper-and-pencil cancellation tasks. They were administered computer-based single- and dual-tasks, requiring to orally name the position of appearance (e.g. left vs. right) of briefly-presented lateralized targets. Patients omitted a consistent number of contralesional targets (≈ 40%) under the single-task condition. When required to perform a concurrent task which recruited additional attentional resources (dual-tasks), patients’ awareness for contralesional hemispace was severely affected, with less than one third of contralesional targets detected (≈ 70% of omissions). In contrast, performance for ipsilesional (right-sided) targets was close to ceiling, showing that the deficit unveiled by computer-based testing selectively affected the contralesional hemispace. We conclude that computer-based, attention-demanding tasks are strikingly more sensitive than cancellation tasks in detecting neglect, because they are relatively immune to compensatory strategies that are often deployed by post-acute patients.

## 1. Introduction

After a brain lesion, the presence and the severity of contralesional neglect is highly variable across patients and is modulated by several factors, including time from onset and assessment methods [1]. In the first days following the occurrence of a neurological insult (commonly, although not necessarily, a stroke), patients often omit targets in the contralesional hemispace on paper-and-pencil cancellation tests. With increasing time from lesion, however, the majority of patients can successfully detect also the most contralesional targets on cancellation tests; this improvement generally applies also to other diagnostic tests of neglect. In the present study, we directly compared, in a group of patients with right-hemisphere damage, the sensitivity of cancellation tests [2], which are the gold standard for neglect assessment [3], with that of a recent computer-based demanding paradigm [4,5] for the detection of contralesional awareness deficits. Our aim was to show that, in the post-acute phase, severe neglect can (re)emerge in apparently intact patients when more sensitive tasks were adopted.

## 2. Method

The sample consisted of 10 right-hemisphere damaged patients (mean age = 67 years, range 40–85, five males) without hemianopia. Mean time from lesion onset was 92 days (median 52). Nine patients suffered a stroke, one a tumour. The sample included eight patients previously described [4,5], who performed the computer-based detection paradigm with the shortest target duration (50 ms; see below) and two new patients.

ISSN 0953-4180/13/$27.50 © 2013 – IOS Press and the authors. All rights reserved
This article is published online with Open Access and distributed under the terms of the Creative Commons Attribution Non-Commercial License

The patients performed the three cancellation subtests of the Behavioural Inattention Test (BIT [2]), a sensitive paper-and-pencil battery for neglect diagnosis. The mean correct responses of the patients were: line cancellation 36/36; letter cancellation 17.5/20 for both left-sided and right-sided targets; stars cancellation 25/27 for left-sided and 25.9/27 for right-sided targets. Left-sided vs. right-sided single target omissions did not differ (Chi-square for each patient and test; all $p$s n.s.) across patients (i.e., *no neglect according to cancellation tests*).

Patients were then tested with the computer-based paradigm, where the main task was to detect briefly presented (50 ms) lateralized target(s), which could occur on the left, on the right, or on both sides. In the single-task (ST) condition, patients had to orally report only the position of the target(s). In the dual-task conditions, patients also performed a second task, visual or auditory, that consumed additional attentional resources (see [4,5] for details). Visual dual task (VDT) required patients to report the identity of a centrally-presented letter before reporting target position. Auditory dual task (ADT) required patients to count by two twice from a heard number before reporting target position.

## 3. Results

For the computer-based test, we analysed only trials where a single unilateral target was presented, disregarding trials with two (bilateral) targets. The percentages of correct detections (or cancelled targets for the cancellation tests) were first entered into a repeated measures ANOVA. Paired t-tests were then performed to compare the percentage of detected stimuli within and across tasks.

### 3.1. Computer-based test

The ANOVA with task (ST, VDT, ADT) and hemispace (left, right) as factors revealed main effects of task, $F(2,18) = 5.1$, $p < 0.05$, and position, $F(1,9) = 32.7$, $p < 0.001$, and an interaction of the two factors, $F(2,18) = 4.6$, $p < 0.05$. The percentage of right-sided (i.e., ipsilesional) single targets correctly reported was 95.5% (collapsed across tasks) and it did not change across tasks (all $p$s ns). The percentage of left (i.e., contralesional) single targets correctly reported was 39.8%. The difference between right-sided and left-sided single targets was significant, $t(9) = 5.7$, $p < 0.001$, indexing a lower number of reported left single targets (i.e., neglect). The percentage of left single targets correctly reported decreased in the dual-task conditions, from 59.7% in the ST to 29.3% in the VDT, $t(9) = 3.18$, $p < 0.05$, and to 30.5% in the ADT, $t(9) = 2.39$, $p < 0.05$. The difference between VDT and ADT was not significant, $t(9) = 0.11$, $p = 0.92$ (see Fig. 1).

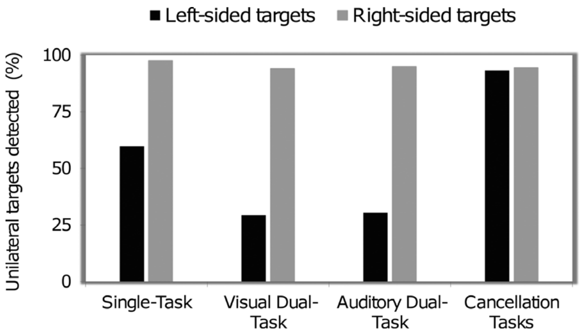

Fig. 1. The percentage of unilateral targets correctly reported is shown as a function of hemispace (left vs. right) and task. Worse performance for left-sided than for right-sided single targets is the signature of neglect; this was evident on all computer-based tasks (Single Task vs. Visual Dual-Task vs. Auditory Dual-Task) but not on the cancellation tests.

### 3.2. Cancellation tests

The ANOVA with task (lines, letters, stars) and hemispace (left, right) as factors revealed only a main effect of task, $F(2, 18) = 5.7$, $p < 0.05$. Importantly, neither the effect of hemispace nor the two-way interaction were significant ($F < 1$).

The difference between cancellation tests and computer-based tests was striking (see Fig. 1): across all cancellation tests (1300 stimuli), the group of patients presented only 9 more omissions on the left (6.9%) than on the right (5.5%). In contrast, across all computer-based tests (about 1800 stimuli), the group of patients presented with 485 more omissions on the left (60.2%) than on the right (4.5%).

## 4. Discussion

Our computer-based test detected severe awareness deficits for contralesional hemispace within a sample of patients in whom cancellation tests failed to reveal any significant ipsilesional bias. The deficit was particularly evident under the more demanding dual-task conditions. Such profound deficits were previously described to result in severe impairments in everyday life [5]. When attentional resources are consumed by a concurrent task, post-acute right-hemisphere damaged patients who perform normally on paper-and-pencil tests frequently show dramatic contralesional awareness deficits [4,5]. The coupling of a multitasking context with brief presentation time results in higher sensitivity [6]. Thus, the assessment of contralesional spatial awareness deficits in the post-acute phase should not only rely on paper-and-pencil tests, in which patients can easily implement compensatory strategies. Computer-based testing seems to be an optimal solution for preventing these strategies and, thus, for increasing the sensitivity of assessment [7,8]. Nevertheless, computer-based testing is seldom, if ever, implemented for the diagnosis of neglect. Thus, many studies on neglect involving a control group of right-hemisphere damaged patients, in which the presence of neglect was excluded by means of paper-and-pencil testing (e.g., [9]), may have encompassed some patients with sub-clinical neglect. Only the use of tests on which no compensation is possible allows clinicians to sensitively determine the true degree of impairment and disability of the patients [10].

Complex everyday life activities (e.g., driving) are much more demanding than the paper-and-pencil tests adopted for neglect diagnosis. Sensitive and demanding dual-task paradigms, such as the one described here, seem to offer a much more appropriate solution for neglect assessment in the post-acute phase and might be particularly useful for a stringent evaluation of rehabilitation outcomes and for inferring performance in complex everyday settings.

## Acknowledgements

This study was supported by a grant from "Compagnia di San Paolo" (Neuroscience Program) for the project "Modulation of attention and spatial awareness by concurrent task demands: Implications for right-hemisphere stroke patients" to M.Z.
